# Estrogen related receptor α (ERRα) a promising target for the therapy of adrenocortical carcinoma (ACC)

**DOI:** 10.18632/oncotarget.4722

**Published:** 2015-07-29

**Authors:** Ivan Casaburi, Paola Avena, Arianna De Luca, Adele Chimento, Rosa Sirianni, Rocco Malivindi, Vittoria Rago, Marco Fiorillo, Francesco Domanico, Carmela Campana, Anna Rita Cappello, Federica Sotgia, Michael P. Lisanti, Vincenzo Pezzi

**Affiliations:** ^1^ Department of Pharmacy, Health and Nutritional Sciences, University of Calabria, Italy; ^2^ The Breakthrough Breast Cancer Research Unit and the Manchester Centre for Cellular Metabolism, Institute of Cancer Sciences, University of Manchester, UK

**Keywords:** ERRα, adrenocortical cancer, mitochondria, ATP depletion

## Abstract

The pathogenesis of the adrenocortical cancer (ACC) involves integration of molecular signals and the interplay of different downstream pathways (i.e. IGFII/IGF1R, β-catenin, Wnt, ESR1). This tumor is characterized by limited therapeutic options and unsuccessful treatments. A useful strategy to develop an effective therapy for ACC is to identify a common downstream target of these multiple pathways. A good candidate could be the transcription factor estrogen-related receptor alpha (ERRα) because of its ability to regulate energy metabolism, mitochondrial biogenesis and signalings related to cancer progression.

In this study we tested the effect of ERRα inverse agonist, XCT790, on the proliferation of H295R adrenocortical cancer cell line. Results from *in vitro* and *in vivo* experiments showed that XCT790 reduced H295R cell growth. The inhibitory effect was associated with impaired cell cycle progression which was not followed by any apoptotic event. Instead, incomplete autophagy and cell death by a necrotic processes, as a consequence of the cell energy failure, induced by pharmacological reduction of ERRα was evidenced.

Our results indicate that therapeutic strategies targeting key factors such as ERRα that control the activity and signaling of bioenergetics processes in high-energy demanding tumors could represent an innovative/alternative therapy for the treatment of ACC.

## INTRODUCTION

Adrenocortical carcinoma (ACC) is a very rare and aggressive disease with a high risk of relapse after radical surgery. Treatment options in advanced, metastatic stages are limited, since cytotoxic chemotherapy options are poor and radiotherapy is mostly ineffective [1]. The drug mitotane (o, p′-dichlorodiphe nyldichloroethane (o, p′-DDD)) with its adrenolytic activity is the only adrenal specific drug that is currently used for ACC treatment. However, toxicity, narrow therapeutic window and side effects are the major limitation to its use as well as therapeutic success [2]. Given the high mortality and aggressiveness of ACC, more effective and specific treatment options are needed. Recently, monoclonal antibodies targeting insulin-like growth factor II (IGFII) receptor (IGF1R) have been tested in clinical trials, however they provided a limited effectiveness in refractory patients [3]. Rationale for targeting IGF1R comes from the observation that IGFII gene is overexpressed in ACC [4]. We have recently demonstrated that IGFII/IGF1R pathway can be activated by the estrogen receptor alpha (ESR1), a gene overexpressed in ACC that mediates estrogen-dependent proliferative effects [5, 6]. ESR1 knock down was more effective than an IGF1R antibody in reducing H295R cell proliferation *in vitro* [5] and the selective estrogen receptor modulator (SERM) tamoxifen prevented the growth of H295R both *in vitro* [7] and as xenografts *in vivo* [5]. Thus, ESR1 could be a promising target to reduce ACC growth.

Indeed, a recent study [8], investigating a large cohort of advanced ACC, confirmed the presence of a large number of potentially targetable molecules involved in ACC progression. These observations confirm that ACC is an extremely heterogeneous disease and that its pathogenesis involves integration of signals and the interplay of downstream pathways. It is currently accepted that these changes are also associated with a profound reprogramming of cellular metabolism [9]. Consequently, one potential strategy to develop an effective therapy for ACC could be the identification of a common downstream target of multiple pathways capable of controlling expression and activity of various bioenergetic factors.

Estrogen Related Receptor α (ERRα) is an orphan member of the nuclear hormone receptor superfamily of transcription factors that has been identified on the basis of its high level of sequence identity to ERα and for which an endogenous ligand has yet to be defined [10]. ERRα functions downstream of the peroxisome proliferator-activated receptor gamma coactivator-1 alpha and beta (PGC-1α and PGC-1β) and regulates the expression of genes involved in energy metabolism and mitochondrial biogenesis such as genes encoding enzymes and proteins of the tricarboxylic acid cycle, pyruvate metabolism, oxidative phosphorylation, and electron transport [11]. Research to understand how changes in cell metabolism promote tumor growth has accelerated in recent years [12]. As a consequence, research has focused on targeting metabolic dependencies of cancer cells, an approach with the potential to have a major impact on patient care. Notably, ERRα has recently been associated with dysregulated cell metabolism and cancer progression. Accordingly, increased expression of ERRα has been shown in several cancerous tissues including breast [13], ovary [14] prostate [15] and colon [16]. Several signaling pathways, also relevant to ACC development have been shown to converge upon and regulate the expression and activity of ERRα together with its coactivators such as PGC-1α and β in others tumor types [17]. Several studies have reported that ERRα inverse agonist XCT-790 [18] can induce cell growth arrest in different tumor cell lines [19, 20]. To date, few studies have investigated the role of ERRα in adrenal gland and ACC. ERRα is expressed in normal adult adrenal and regulates the expression of enzymes involved in steroidogenesis [21]. Moreover, ERRα seems to be more expressed in ACC compared to normal adrenal and adenoma [22].

The aim of this study was to establish if ERRα depletion using XCT790 can induce growth arrest in ACC cells. The data obtained support the hypothesis that ERRα could be a promising target for the treatment of adrenocortical cancer.

## RESULTS

### ERRα inverse agonist XCT790 decreases ERRα protein content and inhibits ACC cells proliferation *in vitro*

**Figure 1:**
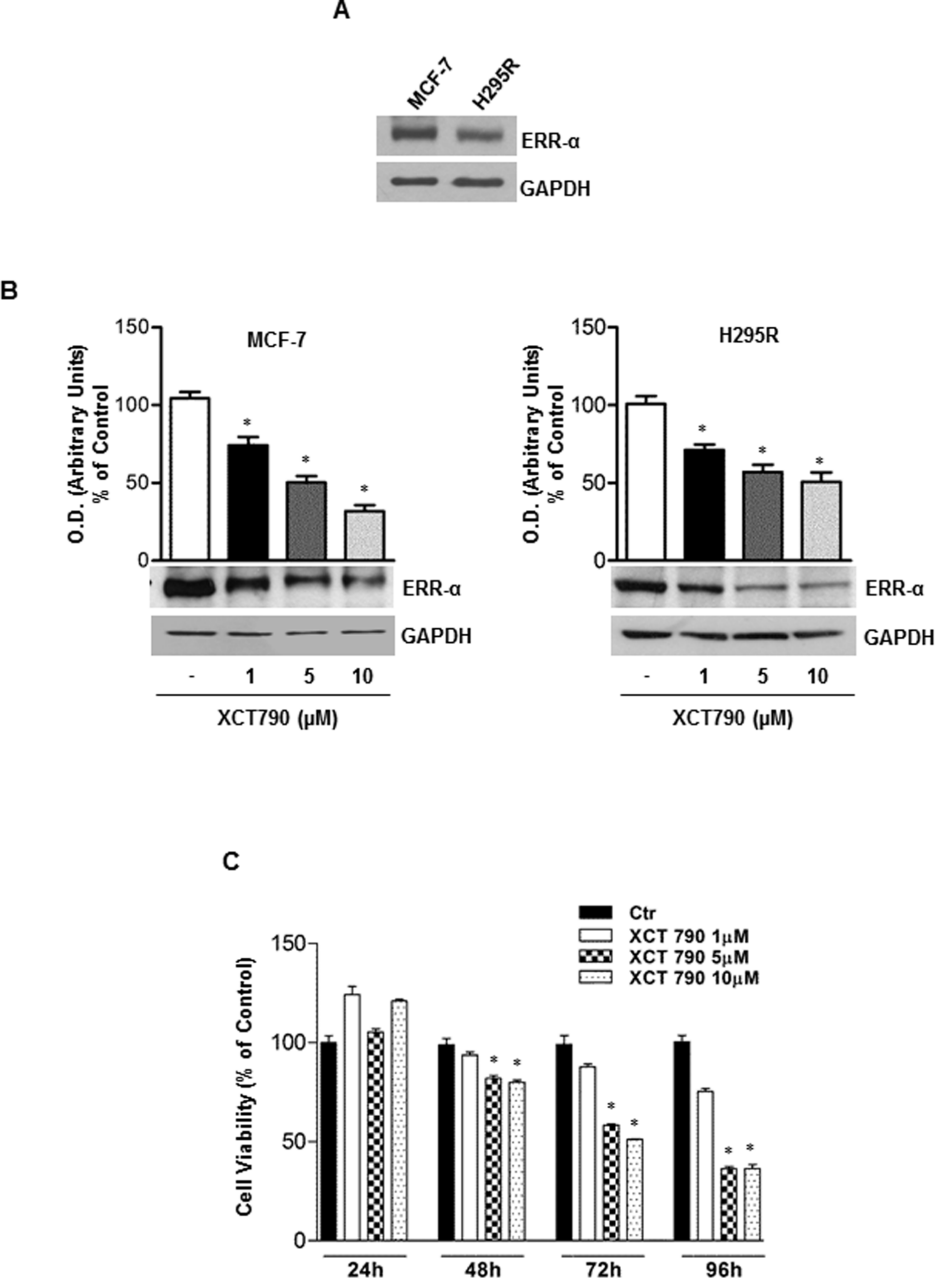
ERRα inverse agonist XCT790 decreases ERRα protein content and H295R cells growth *in vitro* **A.** Western blot analysis of ERRα was performed on 50 μg of total proteins extracted from H295R and MCF-7 cells. Blots are representative of three independent experiments with similar results. (**B.** lower left and right panel), protein extracts from MCF-7 and H295R cells left untreated (−) or treated for 48 h with different doses of XCT790 were resolved by SDS-PAGE and subjected to immunoblot against ERRα. GAPDH served as loading control. (b, upper left and right panel), graphs represent means of ERRα optical density (O.D.) from three independent experiments with similar results normalized to GAPDH content (*p < 0.001 compared to untreated control sample assumed as 100). **C.** Cell viability after XCT790 treatment was measured using MTT assay. Cells were plated in triplicate in 24-well plates and were untreated (Ctr) or treated with increasing concentrations of XCT790 for the indicate times in DMEM supplemented with 2,5% Charcoal-Stripped FBS. Absorbance at 570 nm was measured on a multiwell-plate reader. Cell viability was expressed as a percentage of control, (*p < 0.001).

First, we verified that ERRα is expressed in H295R adrenocortical cancer cells. MCF-7 breast cancer cells were used as positive control [23] (Figure 1A). Moreover, we also demonstrated that in both H295R and MCF-7 cells, XCT790 treatment decreased ERRα protein levels in a dose-dependent manner (Figure 1B). The latter results confirmed the ability of XCT790 to reduce the expression of ERRα most probably by proteasome degradation [23]. Next, we evaluated the effects of different concentrations of ERRα inverse agonist XCT790 on ACC cell growth. Results from MTT assay revealed that XCT790 treatment exerted a dose- and time-dependent inhibition on H295R cell proliferation compared to vehicle-treated cells (Figure 1C). The maximum inhibitory effect on ACC cell proliferation was seen at 10 μM XCT790 that was then used for all the following experiments.

### ERRα inverse agonist XCT790 inhibits ACC cells proliferation *in vivo*

**Figure 2:**
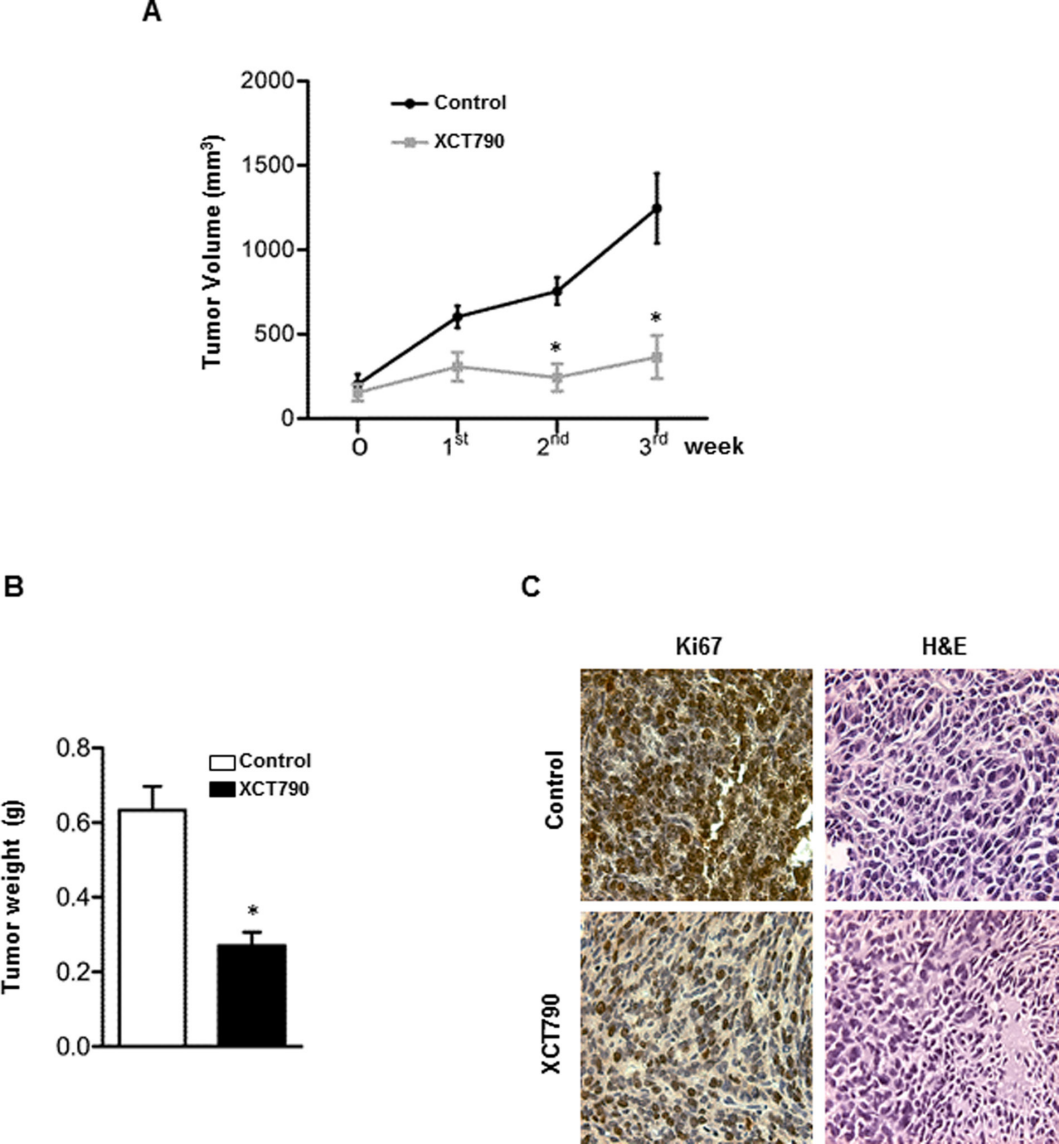
ERRα inverse agonist XCT790 decreases H295R cells proliferation *in vivo* **A.** 6 × 10^6^ H295R cells were injected subcutaneously onto the intrascapular region of immunocompromised mice and the resulting tumors were grown to an average of 200 mm^3^. The animals were randomized to vehicle controls or XCT790 treatment for twenty one days. Tumor volumes were calculated, as indicated in Materials and Methods. Values represent the mean ± SE of measured tumor volume over time in the control group (filled circles, *n* = 10) and in the XCT790-treated group (filled squares, *n* = 10). **B.** After 21 days (3 weeks) tumors were harvested and weighed. Values represent the mean ± SE of measured tumour weight (*n* = 10) *P < 0.05 versus control at the same day of treatment. **C.** Ki67 immunohistochemical and H & E staining: histologic images of H295R explanted from xenograft tumors (magnification X 400).

We next established H295R cell xenograft tumors in immunocompromised mice to investigate the ability of XCT790 to reduce tumor growth *in vivo*. To this aim, H295R cells were injected into the intrascapular region of mice. When tumors reached an average volume of 200 mm^3^, animals were randomized into two groups to be treated with either vehicle or XCT790 (2,5 mg/Kg). As shown in Figure 2A, mice treated with XCT790 displayed a significant tumor growth reduction compared to the vehicle treated control group. Accordingly, tumor reduction upon XCT790 treatment is evidenced both in terms of tumor mass (Figure 2B) and proliferation as seen in Figure 2C, showing a strong decrease in Ki67staining (value score control: 7.2 ± 0,46 (SD); value score XCT790 treated cells: 4.7 ± 0.53* (SD), *p < 0.05).

### ERRα inverse agonist XCT790 blocks G1/S transition of ACC cells without inducing apoptosis

The observed effects of XCT790 on ACC cells proliferation led us to evaluate XCT790 action on H295R cell cycle progression.

**Figure 3:**
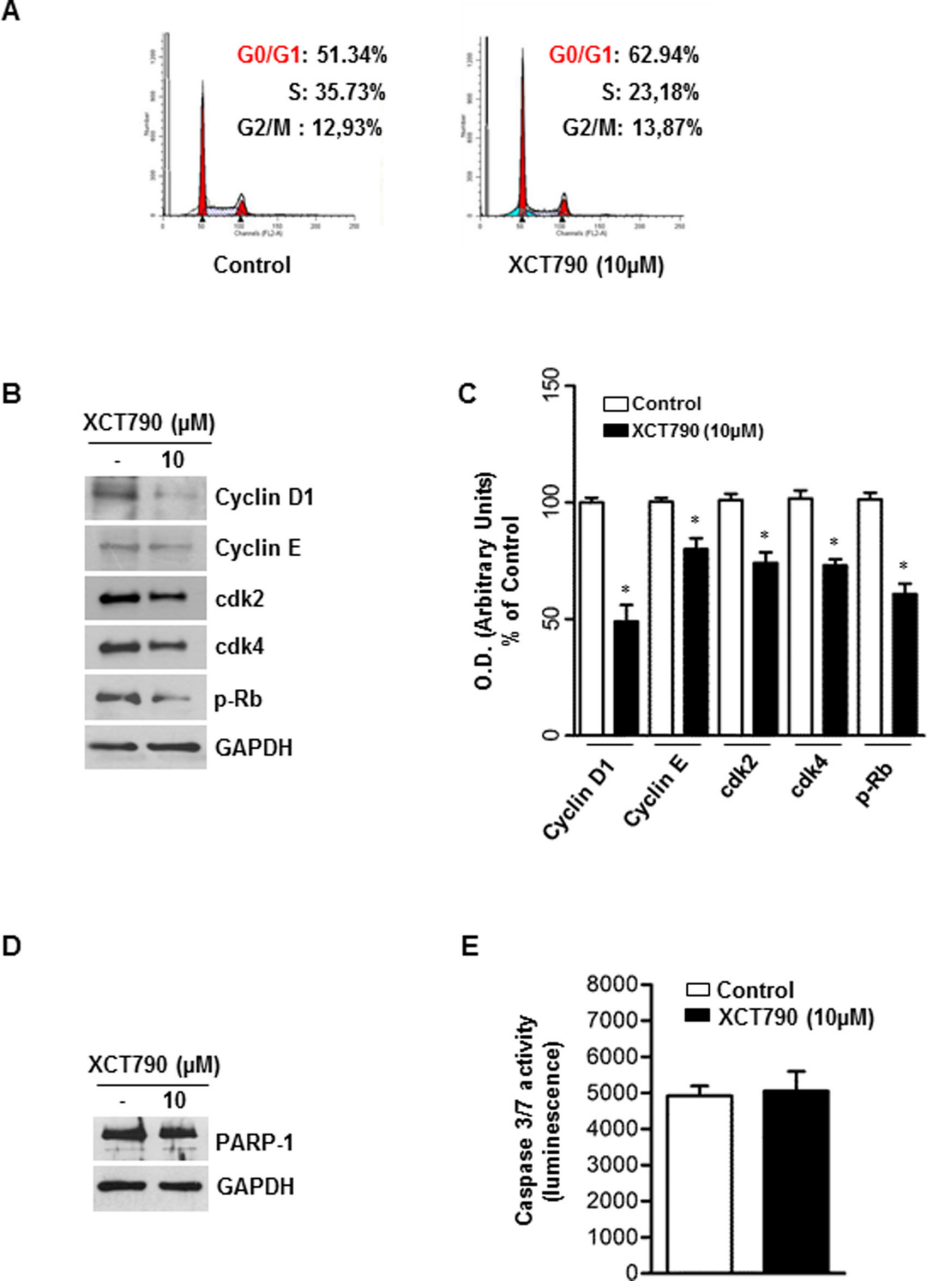
ERRα inverse agonist XCT790 impairs G1/S transition of ACC cells without inducing apoptosis **A.** The distribution of H295R cells in the cycle was determined by Flow Cytometry using Propidium-iodide (PI) stained nuclei. The graph shows the distribution of H295R cell population (%) in the various phases of cell cycle. **B.** Total proteins from H295R cells left untreated (−) or treated with XCT790 for 48 h were resolved by SDS-PAGE and subjected to immunoblot analysis using specific antibodies against human Cyclin D1, Cyclin E, cdk2, cdk4, p-Rb. **C.** Graphs represent means of Cyclin D1, Cyclin E, cdk2, cdk4, p-Rb optical densities (O.D.) from three independent experiments with similar results normalized to GAPDH content, (*p < 0.001 compared to each untreated control assumed as 100); **D.** Total proteins were analyzed by Western blot for PARP-1. Blots are representative of three independent experiments with similar results. GAPDH served as loading control. **E.** Cellular caspase 3/7 activity was determined by Caspase-Glo assay system using the substrate Ac-DEVD-pNA and expressed as relative luminescence units (RLU) of treated cell to untreated control cell. Each column represents the mean ± SD of three independent experiments (*p < 0.001 compared to untreated control sample).

First, by analyzing PI staining with FACSJazz flow cytometer, we investigated whether XCT790 treatment could affect the distribution of cells within the three major phases of the cycle. To this aim, H295R cells were grown for 24 h in 5% CS-FBS and then treated with either vehicle (DMSO) or 10 μM XCT790. 48 hours later, FACS analysis revealed that XCT790 treated cells accumulated in the G0/G1-phase of the cell cycle while the fraction of cells in S phase decreased compared with vehicle treated cells (Figure 3A).

In order to define the molecular mechanisms involved in XCT790-dependent cell cycle arrest, changes in levels of protein involved in cell cycle regulation were investigated by Western blotting analysis. After 48 h treatment, XCT790 reduced Cyclin D1 and Cyclin E protein content while expression levels of CDK2 and CDK4 proteins were unaffected. Consistently with the observed G1/S transition arrest of the cell cycle, Rb protein showed a hypophosphorylated status (Figure 3B–3C). As the analysis of the cell cycle revealed a minimal increase of the sub-G1 fraction (Figure 3A), a known marker of apoptotic events, we next attempted to verify the presence of apoptotic features such as PARP-1cleavage and caspase 3/7 activation, all well-known biochemical markers of programmed cell death. Surprisingly, results from Western blotting analysis for PARP-1 (Figure 3D) and caspase 3/7 activity assay (Figure 3E) clearly showed that XCT790 did not activate an apoptotic pathway.

### XCT-790 decreased mitochondrial mass and function in ACC cells

**Figure 4:**
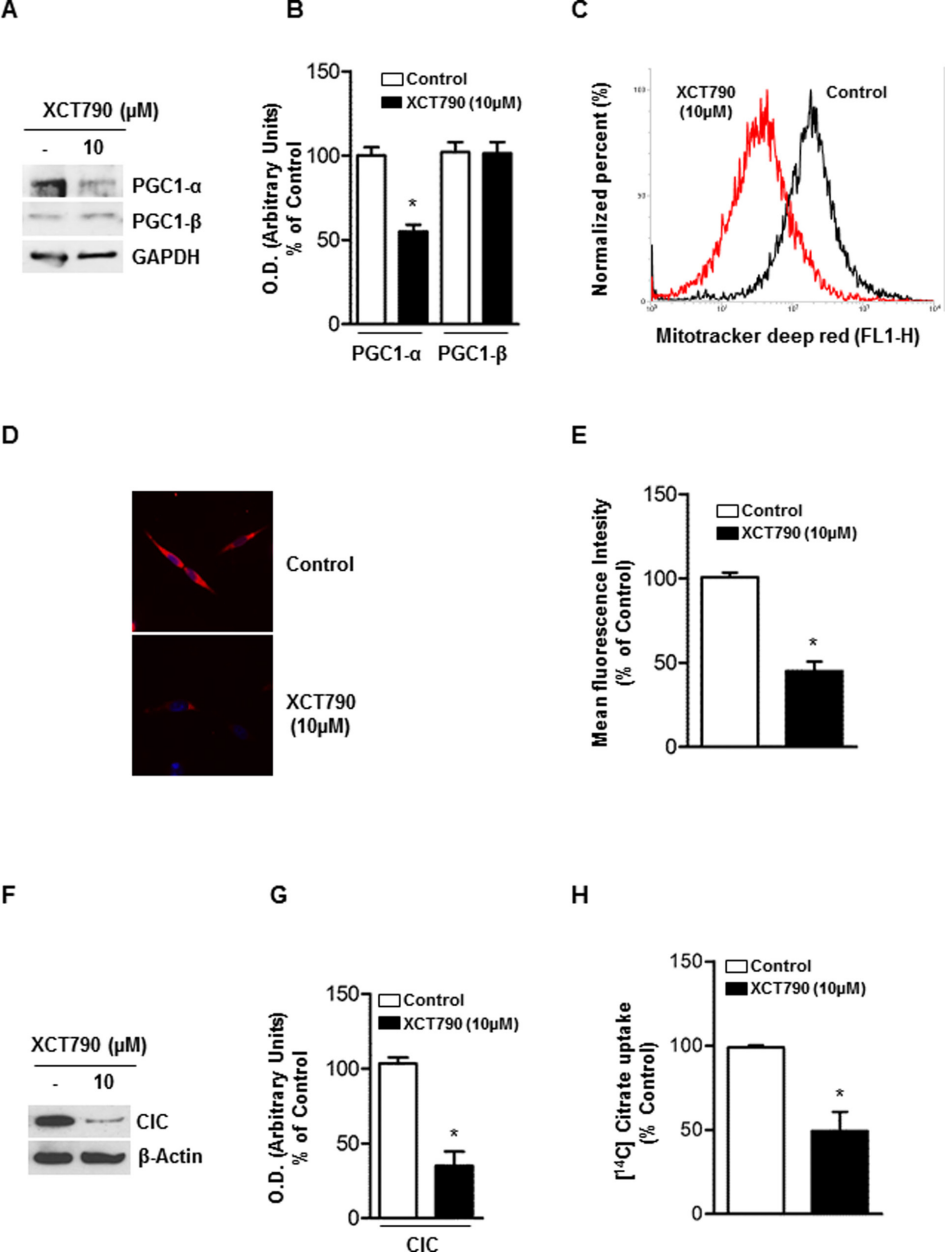
XCT-790 decreases mitochondrial mass and function in H295R cells **A.** Total protein extracts from H295R cells, left untreated (−) or XCT790 treated in 2.5% DCC-FBS medium for 48 h were analyzed by Western blot with antibodies against PGC-1α and PGC-1β. GAPDH was used as loading control. **B.** Graphs represent means of PGC-1α and β optical densities (O.D.) from three independent experiments with similar results normalized to GAPDH content (*p < 0.001 compared to each untreated control sample assumed as 100). **C.** H295R cells were right untreated (control) or treated with XCT790. 48 h later, absorption of MitoTracker deep red FM was determined by FACS analysis. The uptake of MitoTracker was used as an indicator for the mitochondrial mass. **D.** Reduction in mitochondrial mass was further evaluated by fluorescence microscopy of MitoTracker-stained cells. **E.** Quantification of Mito-Tracker fluorescent signal intensity in untreated (control) or XCT790-treated H295R cells was evaluated measuring red fluorescent signal by a fluorescent plate reader (ex. 644; em. 665) *p < 0,001 compared to untreated control sample. **F.** Immunoblots for CIC expression from mitochondrial extracts in untreated (−) or XCT-790 treated H295R cells for 48 h. β-Actin served as loading control. Blots are representative of three independent experiments with similar results. **G.** Graph represent means of CIC density (O.D.) from three independent experiments with similar results normalized to β-Actin content (*p < 0.001 compared to untreated control sample assumed as 100). **H.** CIC activity was measured at 20 min as steady-state levels of citrate/citrate exchange. Transport was started by adding 0.5 mM [^14^C]Citrate to proteoliposomes preloaded internally with 10 mM citrate and reconstituted with mitochondria isolated from untreated H295R cells (Control; white column) and H295R-treated cells (black column). The transport reaction was stopped at 20 minutes. Results are expressed as percentage of the control. The data represent means ± SD of at least three independent experiments.

The activity of ERRα is highly dependent on the presence of coactivator proteins, most notably PGC-1α and PGC-1β [24], both known for their crucial role in regulating energy metabolism and mitochondrial biogenesis [24]. Moreover, it has been observed that XCT790 treatment, causing ERRα proteasome degradation, also down-regulates PGC1-α [24]. Based on these observations, we first checked if XCT790 treatment regulates PGC1-α expression in H295R cells. To this aim, ACC cells were left untreated or treated with 10 μM XCT790 for 48 h. Results from Western blotting showed (Figure 4A–4B) that XCT790 treated cells display a reduced expression of PGC1-α, with no effect on PGC-1β levels. We then asked whether reduced levels of PGC1-α would lead to reduction of mitochondrial mass. To this purpose we treated cells with MitoTracker deep red FM that stains specifically mitochondria independently of their membrane potential. Using flow cytometric analysis (Figure 4C), fluorescent imaging (Figure 4D) and fluorescent plate reader (Figure 4E), we found that XCT790 significantly decreased mitochondrial mass.

The mitochondrial citrate carrier CIC is a protein that belongs to a family of metabolites transporters embedded in the inner mitochondrial membrane [25, 26] and has been recently highlighted as important component in maintaining mitochondrial integrity and bioenergetics in normal and particularly in tumor cells [27]. We used CIC protein expression as a marker of both mitochondrial mass and function and found that XCT790 decreased mitochondrial CIC expression (Figure 4F–4G) as well as its transport activity (Figure 4H) in H295R-treated cells compared to vehicle-treated control cells.

**Figure 5:**
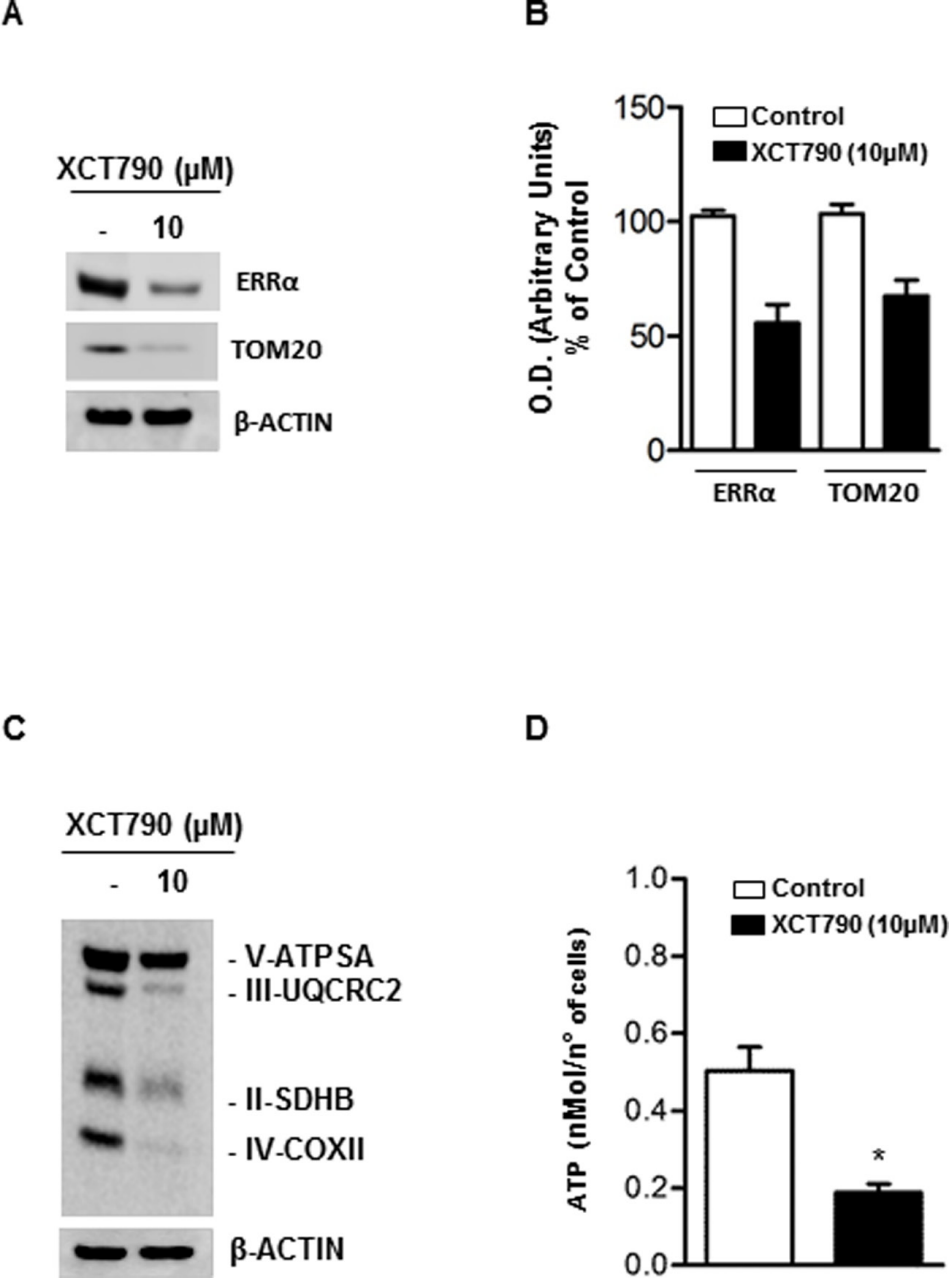
XCT790 decreased OXPHOS protein content and ATP concentration in H295R cells **A.** Total protein extracts from H295R cells, left untreated (−) or treated for 48 h in 2.5% DCC-FBS medium with 10 μM XCT790, were analyzed by Western blot using antibodies against ERRα and TOM20. β-actin was used as loading control. **B.** Graphs represent means of ERRα and TOM20 optical densities (O.D.) from three independent experiments with similar results normalized to β-Actin content (*p < 0.001 compared to each untreated control sample assumed as 100). **C.** Total protein extracts from H295R cells left untreated (−) or treated for 48 h in 2.5% DCC-FBS medium with 10 μM XCT790, were analyzed by Western blot experiments using antibodies against OXPHOS subunits. β-Actin was used as loading control. Blots are representative of three independent experiments with similar results. **D.** ATP concentrations in H295R cells untreated (−) or treated with XCT790 were determined as described in Material and Methods and expressed as nmol/number of cells. Each column represents the mean ± SD of three independent experiments (*p < 0, 001).

To extend these findings, we used immunoblotting to monitor the abundance of a known reliable marker of mitochondrial mass, TOM20, in response to 10 μM XCT790 treatment. We found that XCT790 treated-H295R cells displayed a reduced expression of ERRα, as expected, concomitantly with a drastic decline of TOM20 protein expression (Figure 5A–5B). Similarly, the analysis of the expression of the mitochondrial oxidative pathway (OXPHOS) enzymes showed a substantial reduction of all the complexes (Figure 5C). In agreement with these findings, the reduction in the ATP content reveals a bioenergetics failure induced by XCT790 in treated cells (Figure 5D).

### XCT790 induce cell death by necrosis in ACC cells

**Figure 6:**
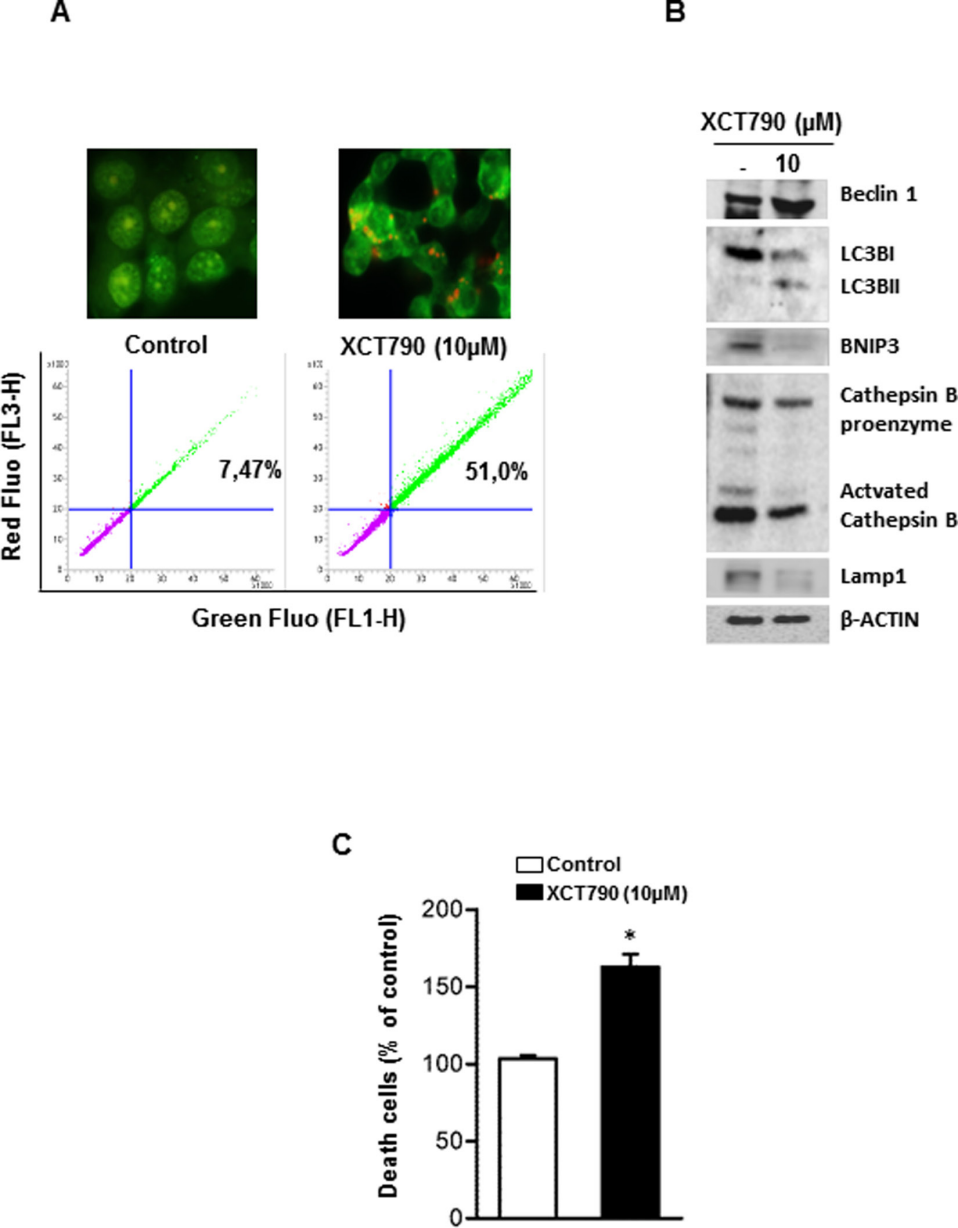
XCT790 induces necrosis in H295R cells **A.** H295R cells were left untreated (control) or treated with XCT790 10 μM. After 48 h, cells were incubated with (1 μg/mL) acridine orange (AO) solution for 30 min at 37°C. Absorption of AO was determined by FACS analysis (lower panel). In the same experimental conditions, treated or untreated H295R cells were stained with acridine orange, mounted and immediately analyzed by fluorescent microscope (upper panel). **B.** Total protein extracts from H295R cells left untreated (−) or treated in 2.5% DCC-FBS medium with XCT790, as indicated, for 48 h were analyzed by Western blot experiments using antibodies against Beclin 1, LC3B, BNIP3, Cathepsin B, Lamp1. β-Actin was used as loading control. Blots are representative of three independent experiments with similar results. **C.** Cell death by necrosis was assessed by Trypan blue-exclusion assay in H295R cells untreated (−) or treated with XCT790. The mean ± SD of three replicates are shown. Cell death was expressed as a percentage of control, (*p < 0.001).

Very recent data revealed that low levels of CIC or its impaired expression induce mitochondrial dysfunction followed by enhanced mitochondrial turnover via autophagy/mitophagy mechanism [27]. Based on this observation and accordingly to our above reported results showing the ability of XCT790 to down-regulate CIC expression in H295R cells, we wanted to verify if autophagic features were detected in our experimental conditions. Autophagy is characterized by acidic vacuoles (AVO) formation, which can be measured by acridine orange (AO) vital staining. AO moves freely to cross biological membranes and accumulates in acidic compartment, where it is seen as bright red fluorescence [28]. As shown in Figure 6A (upper panel), AO vital staining of 48 h XCT790-treated H295R cells showed the accumulation of AVO in the cytoplasm. To quantify the accumulation of the acidific component, we performed FACS analysis of acridine orange-stained cells using FL3 mode (> 650 nm) to quantify the bright red fluorescence and FL1 mode (500–550 nm) for the green fluorescence. As shown in Figure 6A (lower panel), XCT790 treatment raised the strength of red fluorescence from 7,5% to 51%. These results corroborate the observation that XCT790, increases the formation of AVOs which suggests autophagy/mitophagy as possible mechanisms to explain the reduced mitochondrial mass. This latter event could be responsible for the inhibitory effects on cell growth elicited by XCT790 on adrenocortical cancer cells. A careful evaluation of the autophagic/mitophagic process by investigating changes in autophagic markers such as Beclin 1, LC3B, BNIP3 and Cathepsin B (Figure 6B), suggested that XCT790 treatment promotes the initial stages of the autophagic process. This is supported by the evidence of increased Beclin 1 expression and the presence of the cleaved LC3B form [29]. However, autophagy fails to terminate as indicated by decreased BNIP3, Cathepsin B and Lamp1 proteins expression [29]. Therefore, we evaluated XCT790 ability to induce H295R cells death by necrosis. To this aim, Trypan blue exclusion test was performed after 48 h of XCT790 treatment. As shown in Figure 6C, H295R displayed a significant increase in the number of positive stained cells compared to control cells indicating that membrane integrity and permeability were lost accounting for a necrotic event following a bionergetic failure triggered by ERRα depletion.

## DISCUSSION

The molecular heterogeneity and complexity that characterize adrenocortical cancer biology combined with lack of an effective treatment, drive towards the discovery of new therapeutic targets. Advances in the understanding of the molecular pathogenesis of ACC have been made based on studies of gene expression profiling and genetic syndromes associated with the development of ACC [30]. Results from these studies have highlighted the presence of different and important modifications such as somatic TP53 mutations, alterations at 11p15, a chromosomal locus of IGFII, H19 and cyclin-dependent kinase inhibitor 1C, β-catenin accumulation and activation of the Wnt signaling pathway and overexpression of SF-1 protein [30]. Moreover we have recently demonstrated the involvement of ESR1 in ACC cell growth regulation [5]. Genetic modifications and molecular pathways alterations have as a common purpose the survival and proliferation of the transformed phenotype. It is currently accepted that these changes are associated with a concurrent adaptation and reprogramming of cellular metabolism [31]. In this scenario adrenocortical tumors are not an exception and the metabolic receptor ERRα represents a good therapeutic target. In fact, ERRα is a common downstream target of multiple pathways and a key factor in controlling the expression and activity of various bioenergetics processes. Indeed, it has already been observed that high ERRα gene expression correlates with unfavorable clinical outcomes in breast [32] and ovarian cancer [14, 33] and that breast cancer cells exhibiting high ERRα activity are more sensitive to growth inhibition by an ERRα inverse agonist such as XCT790 [34]. Consistent with this findings and with very recent data reporting high ERRα expression in adrenal tumors compared to benign and normal adrenal gland [22], here we report that ERRα is expressed in H295R cells, the most valid cell model to study ACC biology. Moreover, our data show that pharmacological down-regulation of ERRα expression impaired H295R cell proliferation *in vitro* in a dose-dependent fashion. Most importantly, the same inhibitory effect was obtained also in *in vivo* experiments using H295R cells as xenograft model. At the molecular level, the growth inhibition is associated with a G0/G1 cell cycle arrest and by the decreased levels of G1-phase markers such as Cyclin D1 and pRb while CDKs protein levels were unaffected. Noteworthy, cell cycle arrest was not followed by any apoptotic event since we were unable to detect any morphological data (data not shown) or biochemical events such caspase activation and PARP-1 cleavage.

Accumulating data provide evidence that a caspase-independent form of programed cell death such as autophagy can be at play under certain conditions [35]. Therefore we investigated whether the inhibitory effects induced by XCT790 treatment could be linked to autophagy. Our results indicated that XCT790 caused a significant increase in autophagic vesicles. Concomitantly, we observed a drastic reduction in the expression of PGC1-α protein, which plays a key role in mitochondrial biogenesis, and of mitochondrial carrier CIC. The reduction of mitochondrial mass, also confirmed by the reduction of TOM20 protein expression, is followed by a considerable and significant decrease in the ATP concentration. Despite the presence of some autophagic markers such as the up-regulation of Beclin 1 and the cleaved form of LC3 protein, the formation of autophagolysosomes seems to be incomplete as evidenced by the reduction in LAMP1 protein, known to play an important role during the final steps of autophagy process [36]. A possible explanation could be a considerable reduction in the availability of intracellular ATP, required to drive forward the active cell death mechanism including autophagy. On the other hand, we cannot exclude that the observed initial steps of autophagy are a defense cell response to keep cells alive during energy failure to counteract the reduced expression and activity of the master bioenergetic executor ERRα. Moreover, the bioenergetics crisis following treatment with ERRα inverse agonist might be responsible for the loss of plasma membrane integrity, a key signature for a necrotic cell death, allowing the significant increase in the number of Trypan blue stained cells.

However, our most significant finding is that in ACC cells ERRα depletion after XCT790 treatment clearly caused a reduction of mitochondrial function and mass leading to the activation of a number of cellular mechanisms that result in tumor cell death.

It's now well known that mitochondria with its direct involvement in bioenergetics, biosynthesis and cell signaling are mandatory for tumorigenesis. Thus, it's not surprising that many studies have begun to demonstrate that mitochondrial metabolism and signaling is potentially a successful avenue for cancer therapy. Moreover, ACC is (in most cases) characterized by steroids producing/secreting cancer cells highly dependent on functioning mitochondria to ensure steroidogenic processes. For these reasons, strategies using mitochondrial metabolism and signaling as targets should be particularly effective for ACC treatment. Moreover, our current data obtained performing *in vivo* experiments by using H295R cells as xenograft model and according to previous *in vivo* studies performed in breast [37] and leukemia [38] tumor cells also suggest that chemical depletion of ERRα may be specific for high energy demanding cells such as tumor cells without exerting any toxic effect on other tissues.

In conclusion, our study supports the hypothesis that ERRα represents a valid innovative/alternative target for the treatment of adrenocortical cancer.

## MATERIALS AND METHODS

### Cell culture

H295R adrenocortical cancer cells were obtained from Dr. Antonio Stigliano (University of Rome, Italy) and cultured in DMEM/F12 supplemented with 1% ITS Liquid Media Supplement, 10% fetal bovine serum (FBS), 1% glutamine, 2% penicillin/streptomycin (complete medium). MCF7 breast cancer cells were maintained in monolayer cultures DMEM/F12. supplemented with 10% FBS, 1% glutamine, 2% penicillin/streptomycin. Both cell lines were cultured at 37°C in 5% CO_2_ in a humidified atmosphere. All media and supplements were from Sigma-Aldrich, Milano, Italy.

### Western blot analysis

Whole cell lysate were prepared in RIPA buffer (50 mM Tris-HCl, 150 mM NaCl, 1% NP-40, 0.5% sodium deoxycholate, 2 mM sodium fluoride, 2 mM EDTA, 0.1% SDS and a mixture of protease inhibitors) or in ice-cold lysis buffer (10 mM Tris-HCl pH 8, 150 mM NaCl, 1% Triton X-100, 60 mM octylglucoside). Samples were analyzed by 11% SDS-PAGE and blotted onto a nitrocellulose membrane. Blots were incubated overnight at 4°C with anti-ERRα polyclonal antibody, anti-cyclin D1, anti-cyclin E, anti-cdk2, anti-cdk4, anti-p-Rb, anti-PARP, anti-cathepsin B, anti-LAMP1, anti-Tom20 (all from Santa Cruz Biotechnology), anti-Beclin 1 (Novus Biological), anti-LC3B antibody, anti-BNIP3 antibody, Mitoprofile Total OXPHOS Human WB Antibody Cocktail (Abcam) and then incubated with appropriate horseradish peroxidase conjugated secondary antibodies for 1 h at room temperature. The immunoreactive products were detected by the ECL Western blotting detection system (Amersham Pharmacia Biotech, Piscataway, NJ). GAPDH antibody (Santa Cruz Biotechnology) or anti-β-Actin antibody (Sigma-Aldrich) were used as internal control.

### Cell viability assay

H295R cells were seeded in 12-well plates at a density of 1 × 10^5^ cells per well and cultured in complete medium overnight. Before treatment culture medium was switched into in DMEM F-12 supplemented with 2.5% charcoal stripped (CS) FBS and cells were untreated or treated with different concentration of XCT790 (Tocris Bioscience, Bristol, UK) for the indicated time. DMSO (Sigma-Aldrich) was used as vehicle control. Cell viability was measured using MTT assay (Sigma-Aldrich). Each experiment was performed in triplicate and the optical density was measured at 570 nm in a spectrophotometer. Experiments were repeated three times.

### Trypan blue assay

Trypan blue stain was prepared freshly as a 0.4% solution in 0.9% sodium chloride before each experiment. After trypsinization, 20 μl cell suspension was added to 20 μl of Trypan blue solution and mixed thoroughly. Triplicate wells of dye positive cells from untreated or XCT790 treated were counted using a hemocytometer and the experiment was repeated three times.

### Xenograft model

Athymic Nude-Foxn1^nu^ mouse 4–6 weeks old) from Charles River Laboratories [Calco (LC), Italy] were maintained in groups of five or less and quarantined for one week. Mice were kept on a 12 h light/dark cycle with ad libitum access to food and water.

6 × 10^6^ H295R cells suspended in 100 μl of sterile PBS (*Dulbecco's* Phosphate Buffered Saline) and mixed with 100 μl of matrigel, were injected subcutaneously into the intrascapular region of each animal. When tumor size reached a volume of about 200 mm^3^ mice were randomly divided in 2 groups. Animals were injected every other day with vehicle (soy oil) or XCT790 (2,5 mg/Kg) over a 21 day period. Tumors were measured with a caliper every two days, volumes were calculated using the formula *V* = *a b*^*2*^*/2* (*V:volume*; *a* is the length of the long axis, and *b* is the length of the short axis. At the end of the treatment period tumors were harvested and tumor weight and volumes were evaluated. All animal procedures were approved by the local Ethics Committee for Animal Research.

### Immunohistochemical analysis

5 μm thick paraffin-embedded sections were mounted on slides precoated with poly-lysine, and then they were deparaffinized and dehydrated (seven to eight serial sections). Immuno-histochemical experiments were performed using rabbit polyclonal Ki67 primary antibody (Dako, Denmark) at 4°C over-night. Then, a biotinylated goat-anti-rabbit IgG was applied for 1 h at room temperature, followed by avidin biotin-horseradish peroxidase reaction (Vector Laboratories, CA). Immunoreactivity was visualized by using the diaminobenzidine chromogen (Sigma-Aldrich). Counterstaining was carried out with methylene-blue (Sigma-Aldrich). Hematoxylin and eosin Y staining was performed as suggested by the manufacturer (Bio-Optica, Milan, Italy).

### Scoring system

The immunostained slides of tumor samples were evaluated by light microscopy using the Allred Score [39] which combines a proportion score and an intensity score. A proportion score was assigned representing the estimated proportion of positively stained tumor cells (0 = none; 1 = 1/100; 2 = 1/100 to <1/10; 3 = 1/10 to <1/3; 4 = 1/3 to 2/3; 5 = >2/3). An intensity score was assigned by the average estimated intensity of staining in positive cells (0 = none; 1 = weak; 2 = moderate; 3 = strong). Proportion score and intensity score were added to obtain a total score that ranged from 0 to 8. A minimum of 100 cells were evaluated in each slide. Six to seven serial sections were scored in a blinded manner for each sample.

### Cell cycle analysis

H295R cells treated with different doses of XCT790 were fixed, treated with RNase A (20 μg/ml), stained with Propidium iodide (100 μg/ml) (Sigma-Aldrich) and analyzed by Flow Cytometry using BD FACSJazz™ Cell Sorter (Becton, Dickinson and Co) for DNA content and cell cycle status.

### Caspases 3/7 activity assay

Caspases activity was measured with Caspase-Glo Assay Kit (Promega Italia SRL, Milano, Italy) following the manufacturer instruction. The luminescence of each sample was measured in a plate-reading luminometer (Gen5 2.01) with Synergy H1 Hybrid Reader. Each experiment was performed on triplicate wells per condition.

### Mitochondrial mass determination

XCT790 treated or untreated H295R cells were incubated in serum free medium with 200 nM Mitotracker deep red (Invitrogen, USA) for 30 min at 37°C in the dark. After staining, cells were washed twice with cold PBS, trypsinized, centrifuged at 1200 rpm for 5 min and then resuspended in PBS. Absorption of MitoTracker deep red FM was determined by FACS analysis and by fluorescence microscopy. In the same experimental conditions, fluorescent signal intensity was also assessed using a fluorescent plate reader (ex. 644 nM; em. 665 nM).

### Detection of acidic vesicular organelles (AVOs) with acridine orange

H295R cells were cultured on 6 well plates and treated in 2.5% CS-FBS with or without 10 μM XCT790. After 48 h, cells were washed with PBS and stained for 30 min at 37°C with 1 μg/mL acridine orange solution (Sigma-Aldrich). Cells were then washed three times with cold PBS and one drop of mounting solution was added. Cell were observed and imaged by an inverted fluorescence microscope (100X magnification). Accumulation of the acidic vacuoles was also determined by FACS analysis.

### ATP Determination

1 × 10^5^ cells were seeded in 96 white clear bottom multi-well plates in complete medium. Two days later, cells were treated in DMEM F-12 supplemented with 2.5% CS FBS containing 10 μM XCT790. After 48 h, ATP concentrations were determined using the CellTiter-Glo luminescent cell viability assay (Promega) following the manufacturer instruction. Results were normalized to the cell number evaluated by HOECHST staining (Sigma-Aldrich) and expressed as nMol/number of cells.

### Mitochondria reconstitution and transport measurements

The transport activity was carried out as described previously [40]. Briefly, isolated mitochondria from untreated (control) or XCT790 treated H295R cells were solubilized in a buffer containing 3% Triton X, 114, 4 mg/ml cardiolipin, 10 mM Na2SO4, 0.5 mM EDTA, 5 mM PIPES pH 7. The mixture was incubated for 20 min and centrifuged at 138,000 × g for 10 min. The supernatant was incorporated into phospholipid vesicles by cyclic removal of the detergent [41]. The reconstitution mixture consisted of 0.04 mg protein solution, 10% Triton X-114, 10% phospholipids (egg lecithin from Fluka, Milan, Italy) as sonicated liposomes, 10 mM citrate, 0.85 mg/ml cardiolipin (Sigma) and 20 mM PIPES, pH 7.0. The citrate transport was measured after external substrate removal from proteoliposomes on Sephadex G-75 columns, pre-equilibrated with buffer A (50 mM NaCl and 10 mM PIPES, pH 7.0). Transport at 25°C was started by the addition of 0.5 mM [^14^C] citrate (Amersham) to the eluted proteoliposomes and terminated by the addition of 20 mM 1,2,3-benzene-tricarboxylate. Finally, the external radioactivity was removed from the Sephadex G-75 columns, liposomes radioactivity was measured and transport activity was calculated [41].

### Statistics

All experiments were performed at least three times. Data were expressed as mean values ± standard deviation (SD), statistical significance between control and treated samples was analyzed using GraphPad Prism 5.0 (GraphPad Software, Inc.; La Jolla, CA) software. Control and treated groups were compared using the analysis of variance (ANOVA). A comparison of individual treatments was also performed, using Student's t test. Significance was defined as *p* < 0.05.
